# Crystal structure of 1-{(*Z*)-[(2*E*)-3-(4-chloro­phen­yl)-1-phenyl­prop-2-en-1-yl­idene]amino}-3-ethyl­thio­urea

**DOI:** 10.1107/S2056989015023531

**Published:** 2015-12-12

**Authors:** Ming Yueh Tan, Karen A. Crouse, Thahira Begum S. A. Ravoof, Edward R. T. Tiekink

**Affiliations:** a Department of Chemistry, Universiti Putra Malaysia, 43400 Serdang, Malaysia; b Centre for Crystalline Materials, Faculty of Science and Technology, Sunway University, No. 5 Jalan Universiti, 47500 Bandar Sunway, Selangor Darul Ehsan, Malaysia

**Keywords:** crystal structure, hydrogen bonding, thio­semicarbazone

## Abstract

In the title thio­semicarbazone compound, C_18_H_18_ClN_3_S, the CN_3_S residue is almost planar (r.m.s. deviation = 0.0031 Å) and forms dihedral angles of 65.99 (7) and 34.60 (10)° with the phenyl and chloro­benzene rings, respectively; the dihedral angle between the aromatic rings is 85.13 (8)°. The conformation about the C=N bond is *Z*, and that about the C=C bonds is *E*. The imine N and ethyl N atoms are *syn* and are linked by an eth­yl–imine N—H⋯N hydrogen bond. This H atom also forms an inter­molecular hydrogen bond to the thione S atom, resulting in a supra­molecular helical chain propagating along the *b* axis. The chains are consolidated into a three-dimensional architecture by phenyl-C—H⋯Cl contacts and weak π–π inter­actions between centrosymmetrically related chloro­benzene rings [inter-centroid distance = 3.9127 (15) Å].

## Related literature   

For background to the coordination chemistry and applications of metal thio­semicarbazones, see: Dilworth & Hueting (2012 ▸). For the structure of a closely related thio­semicarbazone compound, 1-benzo­thio­phene-2-carbaldehyde 4-ethyl­thio­semicarbazone, with almost planar semicarbazone units (two mol­ecules comprise the asymmetric unit) and *E* conformations for the C=N bonds, see: Kayed *et al.* (2009 ▸).
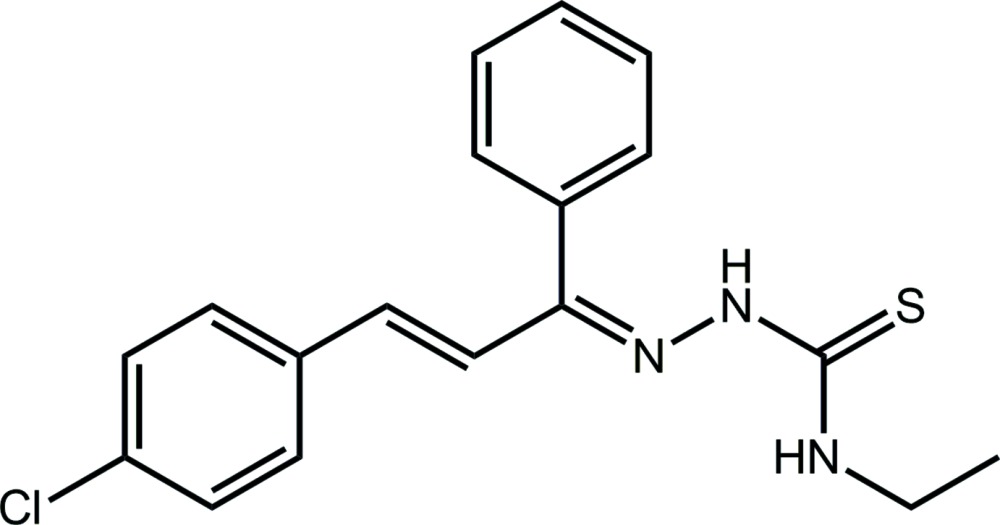


## Experimental   

### Crystal data  

C_18_H_18_ClN_3_S
*M* *_r_* = 343.86
Monoclinic, 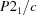

*a* = 10.580 (1) Å
*b* = 12.0438 (9) Å
*c* = 13.9561 (10) Å
β = 90.196 (8)°
*V* = 1778.3 (2) Å^3^
*Z* = 4
Mo *K*α radiation
μ = 0.33 mm^−1^
*T* = 293 K
0.20 × 0.15 × 0.10 mm

### Data collection  

Agilent SuperNova Dual diffractometer with an Atlas detector
Absorption correction: multi-scan (*CrysAlis PRO*; Agilent, 2013 ▸) *T*_min_ = 0.800, *T*_max_ = 1.000
11178 measured reflections
4078 independent reflections
1963 reflections with *I* > 2σ(*I*)
*R*_int_ = 0.051

### Refinement  

*R*[*F* ^2^ > 2σ(*F* ^2^)] = 0.038
*wR*(*F* ^2^) = 0.120
*S* = 1.00
4078 reflections
216 parameters
2 restraints
H-atom parameters constrained
Δρ_max_ = 0.20 e Å^−3^
Δρ_min_ = −0.25 e Å^−3^

### 

Data collection: *CrysAlis PRO* (Agilent, 2013 ▸); cell refinement: *CrysAlis PRO*; data reduction: *CrysAlis PRO*; program(s) used to solve structure: *SHELXS97* (Sheldrick, 2008 ▸); program(s) used to refine structure: *SHELXL2014* (Sheldrick, 2015 ▸); molecular graphics: *ORTEP-3 for Windows* (Farrugia, 2012 ▸) and *DIAMOND* (Brandenburg, 2006 ▸); software used to prepare material for publication: *publCIF* (Westrip, 2010 ▸).

## Supplementary Material

Crystal structure: contains datablock(s) 1, I. DOI: 10.1107/S2056989015023531/hb7555sup1.cif

Structure factors: contains datablock(s) I. DOI: 10.1107/S2056989015023531/hb7555Isup2.hkl

Supporting information file. DOI: 10.1107/S2056989015023531/hb7555Isup3.cml

**Fig. 1.**
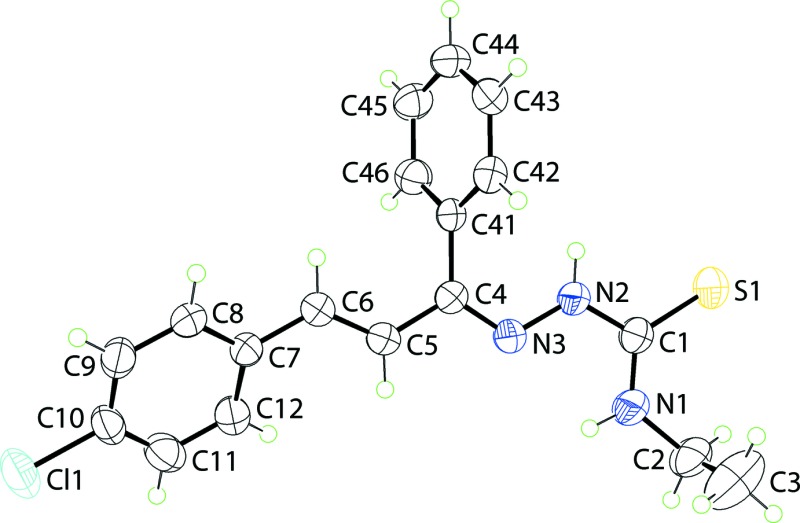
The molecular structure of the title compound showing displacement ellipsoids at the 50% probability level.

**Fig. 2.**
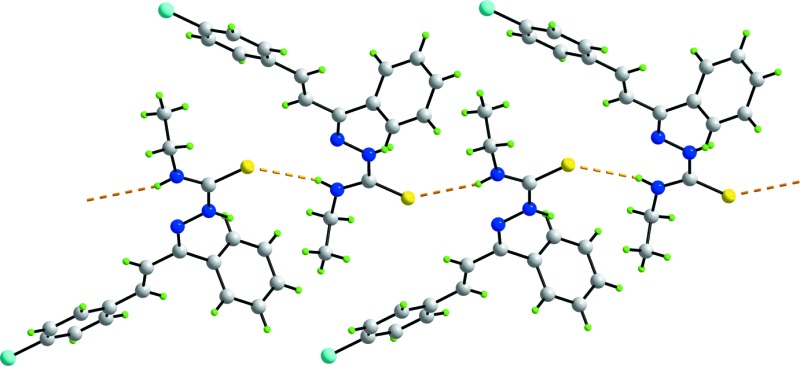
A view of the helical supramolecular chain along the b axis and sustained by N—H···S hydrogen bonds shown as orange dashed lines.

**Fig. 3.**
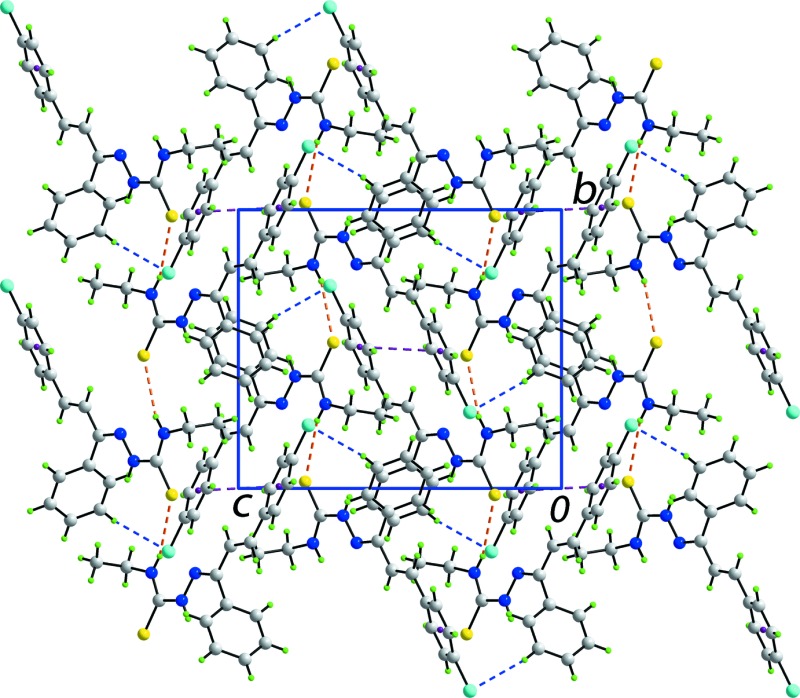
A view of the unit-cell contents in projection down the a axis. The N—H···S (orange), C—H···Cl (blue) and π—π (purple) interactions are shown as dashed lines.

. DOI: 10.1107/S2056989015023531/hb7555fig1.tif
The mol­ecular structure of the title compound showing displacement ellipsoids at the 50% probability level.

b . DOI: 10.1107/S2056989015023531/hb7555fig2.tif
A view of the helical supra­molecular chain along the *b* axis and sustained by N—H⋯S hydrogen bonds shown as orange dashed lines.

a . DOI: 10.1107/S2056989015023531/hb7555fig3.tif
A view of the unit cell contents in projection down the *a* axis. The N—H⋯S (orange), C—H⋯Cl (blue) and π—π (purple) inter­actions are shown as dashed lines.

CCDC reference: 1441045

Additional supporting information:  crystallographic information; 3D view; checkCIF report

## Figures and Tables

**Table 1:** Hydrogen-bond geometry (Å, °)

*D*—H⋯*A*	*D*—H	H⋯*A*	*D*⋯*A*	*D*—H⋯*A*
N1—H1*N*⋯N3	0.88 (2)	2.25 (2)	2.629 (3)	106 (2)
N1—H1*N*⋯S1^i^	0.88 (2)	2.84 (2)	3.693 (2)	165 (2)
C43—H43⋯Cl1^ii^	0.93	2.82	3.708 (3)	160

Symmetry codes: (i) 
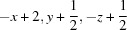
; (ii) 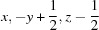.

## supplementary crystallographic information

### S2. Experimental

An ethanol solution (20 ml) of 4-chlorochalcone (0.243 g, 1 mmol) was slowly added to a solution of 4-ethyl-3-thiosemicarbazide (0.119 g, 1 mmol) in absolute ethanol (20 ml), while stirring and heating for about 20 min. The yellow precipitate was filtered, washed with cold ethanol and dried *in vacuo*. Light brown prisms of the title compound were grown at room temperature from the slow evaporation of a solvent mixture of dimethylformamide and acetonitrile; *M*.pt: 154–156 °C.

### S3. Refinement

Carbon-bound H-atoms were placed in calculated positions (C—H = 0.93 to 0.97 Å) and were included in the refinement in the riding model approximation with *U*_iso_(H) = 1.2–1.5*U*_eq_(C). The N—H atom was refined with N—H = 0.88±0.01 Å, and with *U*_iso_(H) = 1.2*U*_eq_(N).

### Crystal data

**Table:**

C_18_H_18_ClN_3_S	*F*(000) = 720
*M**_r_* = 343.86	*D*_x_ = 1.284 Mg m^−^^3^
Monoclinic, *P*2_1_/*c*	Mo *K*α radiation, λ = 0.71073 Å
*a* = 10.580 (1) Å	Cell parameters from 2181 reflections
*b* = 12.0438 (9) Å	θ = 2.9–27.5°
*c* = 13.9561 (10) Å	µ = 0.33 mm^−^^1^
β = 90.196 (8)°	*T* = 293 K
*V* = 1778.3 (2) Å^3^	Prism, light-brown
*Z* = 4	0.20 × 0.15 × 0.10 mm

### Data collection

**Table:**

Agilent SuperNova Dual diffractometer with an Atlas detector	4078 independent reflections
Radiation source: SuperNova (Mo) X-ray Source	1963 reflections with *I* > 2σ(*I*)
Mirror monochromator	*R*_int_ = 0.051
Detector resolution: 10.4041 pixels mm^-1^	θ_max_ = 27.5°, θ_min_ = 2.9°
ω scan	*h* = −13→12
Absorption correction: multi-scan (*CrysAlis PRO*; Agilent, 2013)	*k* = −14→15
*T*_min_ = 0.800, *T*_max_ = 1.000	*l* = −18→18
11178 measured reflections	

### Refinement

**Table:**

Refinement on *F*^2^	Hydrogen site location: mixed
Least-squares matrix: full	H-atom parameters constrained
*R*[*F*^2^ > 2σ(*F*^2^)] = 0.038	*w* = 1/[σ^2^(*F*_o_^2^) + (0.0463*P*)^2^] where *P* = (*F*_o_^2^ + 2*F*_c_^2^)/3
*wR*(*F*^2^) = 0.120	(Δ/σ)_max_ = 0.001
*S* = 1.00	Δρ_max_ = 0.20 e Å^−^^3^
4078 reflections	Δρ_min_ = −0.25 e Å^−^^3^
216 parameters	Extinction correction: *SHELXL2014* (Sheldrick, 2014), Fc^*^=kFc[1+0.001xFc^2^λ^3^/sin(2θ)]^-1/4^
2 restraints	Extinction coefficient: 0.0044 (7)

### Special details

**Table:**

Geometry. All e.s.d.'s (except the e.s.d. in the dihedral angle between two l.s. planes) are estimated using the full covariance matrix. The cell e.s.d.'s are taken into account individually in the estimation of e.s.d.'s in distances, angles and torsion angles; correlations between e.s.d.'s in cell parameters are only used when they are defined by crystal symmetry. An approximate (isotropic) treatment of cell e.s.d.'s is used for estimating e.s.d.'s involving l.s. planes.

### Fractional atomic coordinates and isotropic or equivalent isotropic displacement parameters (Å^2^)

**Table:**

	*x*	*y*	*z*	*U*_iso_*/*U*_eq_	
S1	1.05835 (7)	−0.02341 (6)	0.20649 (5)	0.0606 (2)	
Cl1	0.48690 (8)	0.72659 (6)	0.71676 (5)	0.0754 (3)	
N1	1.0584 (2)	0.19712 (18)	0.23053 (16)	0.0579 (6)	
H1N	1.025 (2)	0.2566 (14)	0.2562 (18)	0.070*	
N2	0.9246 (2)	0.09776 (16)	0.32430 (15)	0.0543 (6)	
H2N	0.900 (2)	0.0320 (11)	0.3439 (17)	0.065*	
N3	0.88627 (18)	0.19560 (15)	0.36594 (14)	0.0484 (5)	
C1	1.0138 (2)	0.0979 (2)	0.25424 (18)	0.0470 (6)	
C2	1.1491 (2)	0.2179 (2)	0.1541 (2)	0.0636 (8)	
H2A	1.2065	0.1553	0.1494	0.076*	
H2B	1.1986	0.2832	0.1699	0.076*	
C3	1.0860 (3)	0.2351 (4)	0.0601 (2)	0.1085 (13)	
H3A	1.0393	0.1696	0.0431	0.163*	
H3B	1.1485	0.2497	0.0121	0.163*	
H3C	1.0292	0.2971	0.0643	0.163*	
C4	0.8004 (2)	0.18918 (18)	0.43172 (16)	0.0420 (6)	
C5	0.7641 (2)	0.29357 (18)	0.47552 (16)	0.0443 (6)	
H5	0.8101	0.3565	0.4592	0.053*	
C6	0.6697 (2)	0.30579 (18)	0.53736 (16)	0.0434 (6)	
H6	0.6254	0.2419	0.5537	0.052*	
C7	0.6281 (2)	0.40943 (18)	0.58244 (16)	0.0409 (6)	
C8	0.5030 (2)	0.42061 (19)	0.61122 (16)	0.0463 (6)	
H8	0.4477	0.3614	0.6026	0.056*	
C9	0.4589 (3)	0.5176 (2)	0.65233 (16)	0.0517 (7)	
H9	0.3749	0.5240	0.6708	0.062*	
C10	0.5415 (3)	0.60409 (19)	0.66531 (16)	0.0504 (7)	
C11	0.6661 (3)	0.5955 (2)	0.63902 (18)	0.0576 (7)	
H11	0.7209	0.6548	0.6489	0.069*	
C12	0.7099 (3)	0.49840 (19)	0.59786 (18)	0.0529 (7)	
H12	0.7943	0.4925	0.5804	0.063*	
C41	0.7411 (2)	0.08309 (17)	0.46264 (17)	0.0391 (6)	
C42	0.6659 (2)	0.02282 (19)	0.39972 (17)	0.0446 (6)	
H42	0.6525	0.0488	0.3377	0.054*	
C43	0.6108 (2)	−0.07542 (19)	0.42866 (19)	0.0506 (7)	
H43	0.5608	−0.1156	0.3860	0.061*	
C44	0.6294 (2)	−0.1139 (2)	0.5200 (2)	0.0550 (7)	
H44	0.5916	−0.1799	0.5394	0.066*	
C45	0.7037 (2)	−0.0552 (2)	0.58311 (19)	0.0553 (7)	
H45	0.7163	−0.0817	0.6450	0.066*	
C46	0.7597 (2)	0.0428 (2)	0.55490 (17)	0.0491 (6)	
H46	0.8101	0.0821	0.5978	0.059*	

### Atomic displacement parameters (Å^2^)

**Table:**

	*U*^11^	*U*^22^	*U*^33^	*U*^12^	*U*^13^	*U*^23^
S1	0.0614 (5)	0.0544 (4)	0.0661 (5)	0.0084 (3)	0.0204 (4)	−0.0080 (4)
Cl1	0.1222 (7)	0.0502 (4)	0.0539 (4)	0.0289 (4)	0.0068 (4)	−0.0074 (3)
N1	0.0567 (14)	0.0503 (14)	0.0670 (15)	−0.0007 (11)	0.0263 (12)	0.0014 (12)
N2	0.0638 (14)	0.0380 (12)	0.0614 (13)	0.0005 (10)	0.0292 (11)	−0.0016 (11)
N3	0.0531 (12)	0.0375 (11)	0.0548 (12)	0.0017 (9)	0.0176 (11)	−0.0023 (10)
C1	0.0433 (14)	0.0470 (16)	0.0506 (14)	0.0010 (11)	0.0098 (12)	−0.0017 (13)
C2	0.0500 (16)	0.0699 (18)	0.0710 (19)	−0.0065 (13)	0.0207 (15)	0.0061 (16)
C3	0.078 (2)	0.172 (4)	0.076 (2)	−0.021 (2)	0.006 (2)	0.031 (3)
C4	0.0426 (14)	0.0386 (14)	0.0450 (14)	−0.0014 (10)	0.0090 (12)	−0.0001 (12)
C5	0.0482 (15)	0.0349 (13)	0.0500 (14)	−0.0011 (10)	0.0094 (12)	−0.0001 (11)
C6	0.0465 (14)	0.0378 (14)	0.0459 (14)	−0.0015 (10)	0.0062 (12)	−0.0017 (11)
C7	0.0491 (15)	0.0368 (13)	0.0368 (13)	0.0022 (11)	0.0066 (11)	0.0014 (11)
C8	0.0537 (16)	0.0411 (14)	0.0442 (14)	0.0000 (11)	0.0115 (12)	0.0051 (12)
C9	0.0611 (17)	0.0494 (16)	0.0448 (15)	0.0155 (13)	0.0141 (13)	0.0075 (13)
C10	0.079 (2)	0.0373 (14)	0.0348 (13)	0.0122 (13)	0.0034 (13)	−0.0021 (12)
C11	0.0728 (19)	0.0446 (16)	0.0555 (16)	−0.0044 (13)	−0.0035 (15)	−0.0065 (14)
C12	0.0543 (17)	0.0489 (16)	0.0555 (16)	−0.0019 (12)	0.0096 (13)	−0.0080 (13)
C41	0.0404 (13)	0.0342 (13)	0.0427 (14)	0.0028 (10)	0.0110 (11)	−0.0028 (11)
C42	0.0470 (15)	0.0450 (14)	0.0419 (13)	0.0016 (11)	0.0075 (12)	−0.0019 (12)
C43	0.0538 (16)	0.0433 (14)	0.0547 (17)	−0.0066 (12)	0.0098 (13)	−0.0102 (13)
C44	0.0603 (17)	0.0396 (14)	0.0652 (18)	−0.0061 (12)	0.0154 (15)	0.0042 (14)
C45	0.0653 (18)	0.0503 (16)	0.0502 (15)	0.0011 (13)	0.0076 (14)	0.0104 (14)
C46	0.0532 (16)	0.0472 (15)	0.0469 (15)	−0.0046 (12)	0.0003 (12)	−0.0003 (13)

### Geometric parameters (Å, º)

**Table:**

S1—C1	1.674 (2)	C7—C8	1.392 (3)
Cl1—C10	1.740 (2)	C7—C12	1.393 (3)
N1—C1	1.327 (3)	C8—C9	1.383 (3)
N1—C2	1.459 (3)	C8—H8	0.9300
N1—H1N	0.874 (10)	C9—C10	1.372 (4)
N2—C1	1.361 (3)	C9—H9	0.9300
N2—N3	1.376 (2)	C10—C11	1.373 (4)
N2—H2N	0.878 (10)	C11—C12	1.384 (3)
N3—C4	1.296 (2)	C11—H11	0.9300
C2—C3	1.484 (4)	C12—H12	0.9300
C2—H2A	0.9700	C41—C42	1.388 (3)
C2—H2B	0.9700	C41—C46	1.389 (3)
C3—H3A	0.9600	C42—C43	1.380 (3)
C3—H3B	0.9600	C42—H42	0.9300
C3—H3C	0.9600	C43—C44	1.370 (3)
C4—C5	1.450 (3)	C43—H43	0.9300
C4—C41	1.488 (3)	C44—C45	1.375 (4)
C5—C6	1.330 (3)	C44—H44	0.9300
C5—H5	0.9300	C45—C46	1.379 (3)
C6—C7	1.466 (3)	C45—H45	0.9300
C6—H6	0.9300	C46—H46	0.9300
			
C1—N1—C2	124.9 (2)	C9—C8—C7	121.6 (2)
C1—N1—H1N	119.5 (17)	C9—C8—H8	119.2
C2—N1—H1N	115.1 (17)	C7—C8—H8	119.2
C1—N2—N3	120.56 (18)	C10—C9—C8	118.7 (2)
C1—N2—H2N	115.5 (16)	C10—C9—H9	120.6
N3—N2—H2N	123.6 (16)	C8—C9—H9	120.6
C4—N3—N2	117.15 (18)	C9—C10—C11	121.3 (2)
N1—C1—N2	115.3 (2)	C9—C10—Cl1	119.1 (2)
N1—C1—S1	125.86 (17)	C11—C10—Cl1	119.6 (2)
N2—C1—S1	118.80 (17)	C10—C11—C12	119.8 (2)
N1—C2—C3	112.0 (2)	C10—C11—H11	120.1
N1—C2—H2A	109.2	C12—C11—H11	120.1
C3—C2—H2A	109.2	C11—C12—C7	120.4 (2)
N1—C2—H2B	109.2	C11—C12—H12	119.8
C3—C2—H2B	109.2	C7—C12—H12	119.8
H2A—C2—H2B	107.9	C42—C41—C46	118.9 (2)
C2—C3—H3A	109.5	C42—C41—C4	120.5 (2)
C2—C3—H3B	109.5	C46—C41—C4	120.7 (2)
H3A—C3—H3B	109.5	C43—C42—C41	120.3 (2)
C2—C3—H3C	109.5	C43—C42—H42	119.8
H3A—C3—H3C	109.5	C41—C42—H42	119.8
H3B—C3—H3C	109.5	C44—C43—C42	120.2 (2)
N3—C4—C5	115.73 (19)	C44—C43—H43	119.9
N3—C4—C41	123.63 (19)	C42—C43—H43	119.9
C5—C4—C41	120.64 (18)	C43—C44—C45	120.1 (2)
C6—C5—C4	124.7 (2)	C43—C44—H44	119.9
C6—C5—H5	117.6	C45—C44—H44	119.9
C4—C5—H5	117.6	C44—C45—C46	120.2 (2)
C5—C6—C7	126.8 (2)	C44—C45—H45	119.9
C5—C6—H6	116.6	C46—C45—H45	119.9
C7—C6—H6	116.6	C45—C46—C41	120.2 (2)
C8—C7—C12	118.1 (2)	C45—C46—H46	119.9
C8—C7—C6	119.6 (2)	C41—C46—H46	119.9
C12—C7—C6	122.3 (2)		
			
C1—N2—N3—C4	179.6 (2)	C9—C10—C11—C12	0.5 (4)
C2—N1—C1—N2	−176.5 (2)	Cl1—C10—C11—C12	−179.8 (2)
C2—N1—C1—S1	3.9 (4)	C10—C11—C12—C7	0.3 (4)
N3—N2—C1—N1	−0.2 (4)	C8—C7—C12—C11	−1.2 (4)
N3—N2—C1—S1	179.39 (19)	C6—C7—C12—C11	178.8 (2)
C1—N1—C2—C3	87.9 (4)	N3—C4—C41—C42	−65.7 (3)
N2—N3—C4—C5	178.8 (2)	C5—C4—C41—C42	114.7 (2)
N2—N3—C4—C41	−0.9 (4)	N3—C4—C41—C46	114.5 (3)
N3—C4—C5—C6	173.2 (2)	C5—C4—C41—C46	−65.2 (3)
C41—C4—C5—C6	−7.1 (4)	C46—C41—C42—C43	0.0 (3)
C4—C5—C6—C7	−179.0 (2)	C4—C41—C42—C43	−179.9 (2)
C5—C6—C7—C8	152.8 (2)	C41—C42—C43—C44	0.3 (3)
C5—C6—C7—C12	−27.2 (4)	C42—C43—C44—C45	−0.4 (4)
C12—C7—C8—C9	1.3 (3)	C43—C44—C45—C46	0.1 (4)
C6—C7—C8—C9	−178.7 (2)	C44—C45—C46—C41	0.2 (4)
C7—C8—C9—C10	−0.4 (4)	C42—C41—C46—C45	−0.2 (3)
C8—C9—C10—C11	−0.5 (4)	C4—C41—C46—C45	179.6 (2)
C8—C9—C10—Cl1	179.87 (18)		

### Hydrogen-bond geometry (Å, º)

**Table:**

*D*—H···*A*	*D*—H	H···*A*	*D*···*A*	*D*—H···*A*
N1—H1*N*···N3	0.88 (2)	2.25 (2)	2.629 (3)	106 (2)
N1—H1*N*···S1^i^	0.88 (2)	2.84 (2)	3.693 (2)	165 (2)
C43—H43···Cl1^ii^	0.93	2.82	3.708 (3)	160

Symmetry codes: (i) −*x*+2, *y*+1/2, −*z*+1/2; (ii) *x*, −*y*+1/2, *z*−1/2.
