# Supplementary figures and images for: Crystal structure of 1-{(Z)-[(2E)-3-(4-chloro­phen­yl)-1-phenyl­prop-2-en-1-yl­idene]amino}-3-ethyl­thio­urea
Source: Acta Crystallogr E Crystallogr Commun. 2015 Dec 12;71(Pt 12):o1047–8. doi: 10.1107/S2056989015023531 (PMC4719972; doi:10.1107/S2056989015023531)

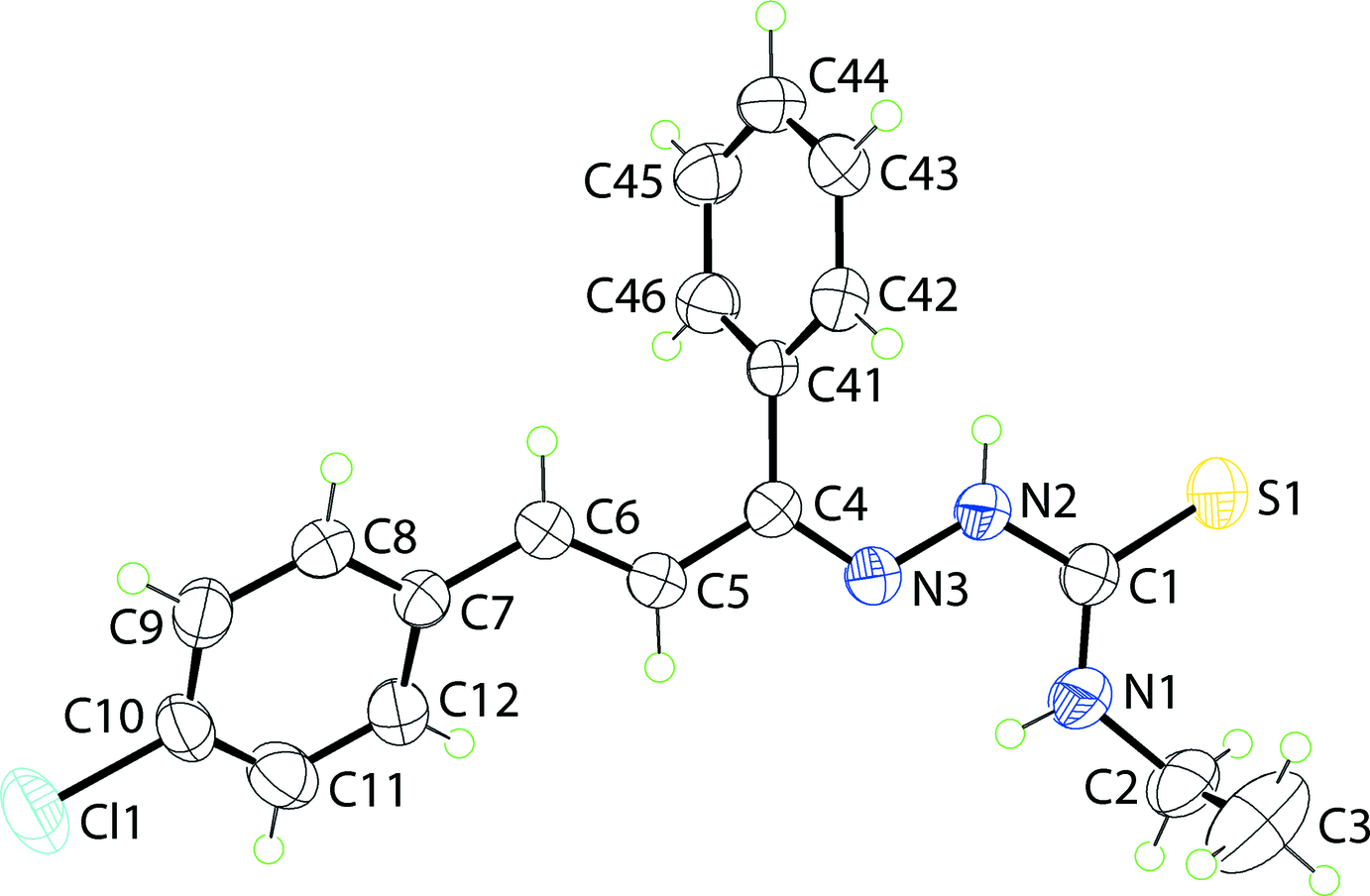

Supplement: Supplementary file 4 [file e-71-o1047-fig1.tif]

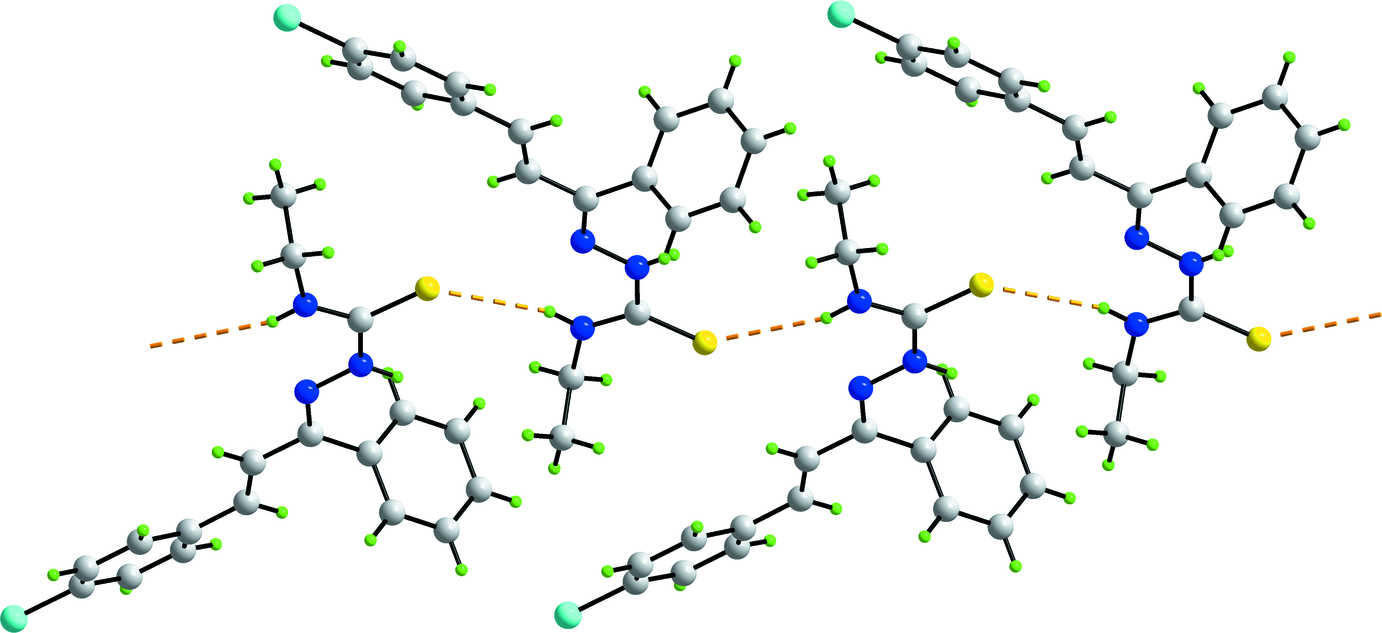

Supplement: Supplementary file 5 [file e-71-o1047-fig2.tif]

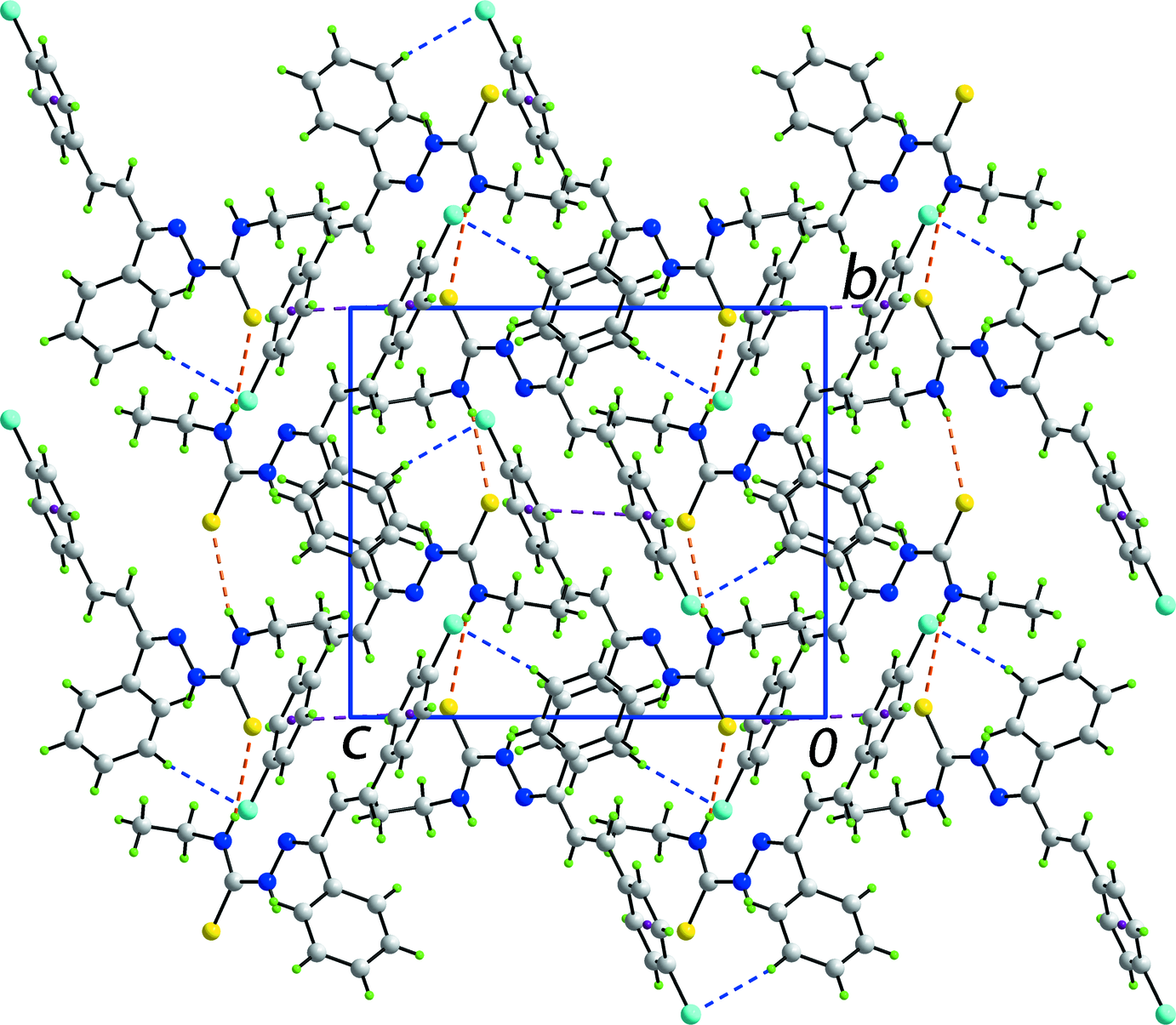

Supplement: Supplementary file 6 [file e-71-o1047-fig3.tif]
